# Identification of neuraminidase inhibitors against dual H274Y/I222R mutant strains

**DOI:** 10.1038/s41598-017-12101-3

**Published:** 2017-09-26

**Authors:** Kai-Cheng Hsu, Hui-Chen Hung, Wei-Chun HuangFu, Tzu-Ying Sung, Tony Eight Lin, Ming-Yu Fang, I-Jung Chen, Nikhil Pathak, John T.-A. Hsu, Jinn-Moon Yang

**Affiliations:** 1 Graduate Institute of Cancer Biology and Drug Discovery, College of Medical Science and Technology, Taipei Medical University, Taipei, Taiwan; 2 Institute of Biotechnology and Pharmaceutical Research, National Health Research Institutes, Miaoli, Taiwan; 3 Institute of Bioinformatics and Systems Biology, National Chiao Tung University, Hsinchu, Taiwan; 4 TIGP-Bioinformatics, Institute of Information Science, Academia Sinica, Taipei, Taiwan; 5 Department of Biological Science and Technology, National Chiao Tung University, Hsinchu, Taiwan

## Abstract

Influenza is an annual seasonal epidemic that has continually drawn public attentions, due to the potential death toll and drug resistance. Neuraminidase, which is essential for the spread of influenza virus, has been regarded as a valid target for the treatment of influenza infection. Although neuraminidase drugs have been developed, they are susceptible to drug-resistant mutations in the sialic-binding site. In this study, we established computational models (site-moiety maps) of H1N1 and H5N1 to determine properties of the 150-cavity, which is adjacent to the drug-binding site. The models reveal that hydrogen-bonding interactions with residues R118, D151, and R156 and van der Waals interactions with residues Q136, D151, and T439 are important for identifying 150-cavitiy inhibitors. Based on the models, we discovered three new inhibitors with IC_50_ values <10 μM that occupies both the 150-cavity and sialic sites. The experimental results identified inhibitors with similar activities against both wild-type and dual H274Y/I222R mutant neuraminidases and showed little cytotoxic effects. Furthermore, we identified three new inhibitors situated at the sialic-binding site with inhibitory effects for normal neuraminidase, but lowered effects for mutant strains. The results suggest that the new inhibitors can be used as a starting point to combat drug-resistant strains.

## Introduction

Influenza virus causes severe respiratory illness and death each year. In recent years, outbreaks of avian influenza H5N1 virus have attracted public attentions^1–3^. In addition, a new strain of influenza H1N1 virus, which originated in swine, has rapidly spread to many countries^4^. Two glycoproteins (haemagglutinin (H) and neuraminidase (NA)), play an important role in viral replication in host cells. Haemagglutinin will initiate virus infection by binding to the sialic acid receptor. Afterwards, neuraminidase will facilitate the release of newly replicated viruses for infection to other cells^5,6^. Although vaccination is the primary strategy to prevent influenza infection, vaccines are often inadequate due to the high mutation rate of influenza viral antigens^7^. Therefore, other targets for the influenza virus are needed.

Most neuraminidase inhibitors show promising results in reducing overall mortality with prompt treatment and its usage has since been on the rise^8,9^. Structure-based drug designs have been applied to successfully identify four drugs, zanamivir (Relenza), oseltamivir (Tamiflu)^10^, peramivir (Rapivab)^11^, and laninamivir^12^. These four drugs were designed based on the transition state of sialic acid^13–15^, and are generally used for the therapy of influenza virus infections^10^. However, the emergence of drug-resistant NA strains has been reported for these drugs during treatment^16–21^. Currently, there is a debate on the effectiveness of oseltamivir, which has been shown to reduce symptoms in adults, but did not reduce the number of people with flue complications^22^. The World Health Organization has downgraded oseltamivir from the list of core to complementary drugs^23^. As a result, there is a growing need for developing new antiviral inhibitors to treat influenza virus infections.

The structures of NA can be separated into two subtypes, group-1 (N1, N4, N5 and N8) and group-2 (N2, N3, N6, N7 and N9), according to their phylogenetic distances^5^. Group-1 generally exhibits a cavity known as the 150-cavity, so called due to the loop containing residues 147-152^24^. This cavity has two distinct conformations, open and closed. Recently, the crystal structures of group-1 NAs reveal that the 150-loop is able to maintain an open form to create a ‘150-cavity’ adjacent to the sialic acid binding site, while group-2 NAs contains a closed conformation^5,25^. However, the 150-loop of group-2 NAs may be induced to form an open conformation by inhibitors, due to a high sequence similarity in the 150-loops between group-1 and group-2^5^. It has been shown that targeting the 150-cavity can enhance antiviral specificity and potency against group-1 NAs^26^. Inhibitors targeting the 150-cavity are considered useful for circumventing zanamivir and oseltamivir resistant influenza viruses, which contain mutations within the sialic acid binding site or mutations outside of the binding site^16,17^. The mutations located outside of the binding site can also influence the size of binding site structure^27^. Therefore, developing drugs with different action mechanisms is required for the treatment of drug-resistant NAs.

Resistance to neuraminidase inhibitors, such as zanamivir and oseltamivir, is becoming an emergent issue. According to previous studies, the H1N1 IC_50_ values typically differ by at least 200 folds between wildtype (IC_50_ value between 500–1000 nM) and mutant strains (IC_50_ value between 0.9–2.0 nM)^16–19^. These strains include an oseltamivir carboxylate-resistant strain, where a tyrosine replaces histidine at position 274 in NA, and a zanamivir-resistant strain, where an arginine replaces isoleucine at position 222^19,28^. The mutations, which are found within the sialic acid site, have also yielded a multiple drug-resistant (MDR) strain, which further reduces zanamivir and oseltamivir potency^29^. Since known drugs will interact with these mutation residues, they are more susceptible to becoming inactive against these influenza strains. Thus, it is of great importance to identify anti-influenza NA agents that exploit the 150-cavity.

To identify potential inhibitors, our study virtually screened compounds from the National Cancer Institute (NCI) database. Previous virtual screening approaches have been applied to NCI molecular library^30,31^. Cheng *et al*. identified potential novel antiviral compounds from NCI library^30^, which need to be further validated by assays. Hoffmann *et al*. discovered a series of diazenylaryl sulfonic acids as NA inhibitors, which inhibited N1 NA with drug-resistant mutations^31^, including H274Y, N294S, Y155H, Q136L, I427Q and I427M. However, the physiochemical properties of the 150-cavity were not characterized, nor an analysis based on a dual H274Y/I222R mutation of an influenza virus. The H274Y/I222R mutation have shown an increased resistance to zanamivir, oseltamivir, and peramivir^19,29^. To solve these issues, our study established a site-moiety map^32^ to elucidate properties of the binding site and discover new inhibitors for dual H274Y/I222R mutant NA.

We used a site-moiety map (SiMMap) to analyze a protein binding site to identify anchors, which are often key binding environments^32^. Each anchor contains a binding pocket, which is composed of conserved interacting residues within the binding site that include moiety preferences and interactions (electrostatic (E), hydrogen-bonding (H), or van der Waals (V)). A compound that matches anchors has a greater potential of inhibiting the target protein. In addition, our experimental results show that an anchor is often a hot spot and the SiMMap can help assemble potential leads by optimal steric, hydrogen-bonding, and electronic moieties^32^. With these models, we are able to identify inhibitors with novel scaffolds. Such inhibitors are able to provide an opportunity to design anti-resistance inhibitors against MDR NA strains.

In this study, we virtually screened the NCI database as well as in-house compounds to identify potential NA inhibitors. A SiMMap was established to determine the most favorable binding pockets within the active site. Our method unveiled six new NA inhibitors. These results confirm the benefits of the screening strategy and creation of the SiMMap to identify novel inhibitors. This provides a beneficial strategy for potentially developing new drugs to combat drug-resistant pathogens or human diseases.

## Results

### Overview of site-moiety map for discovering neuraminidase inhibitors

**Figure 1:**
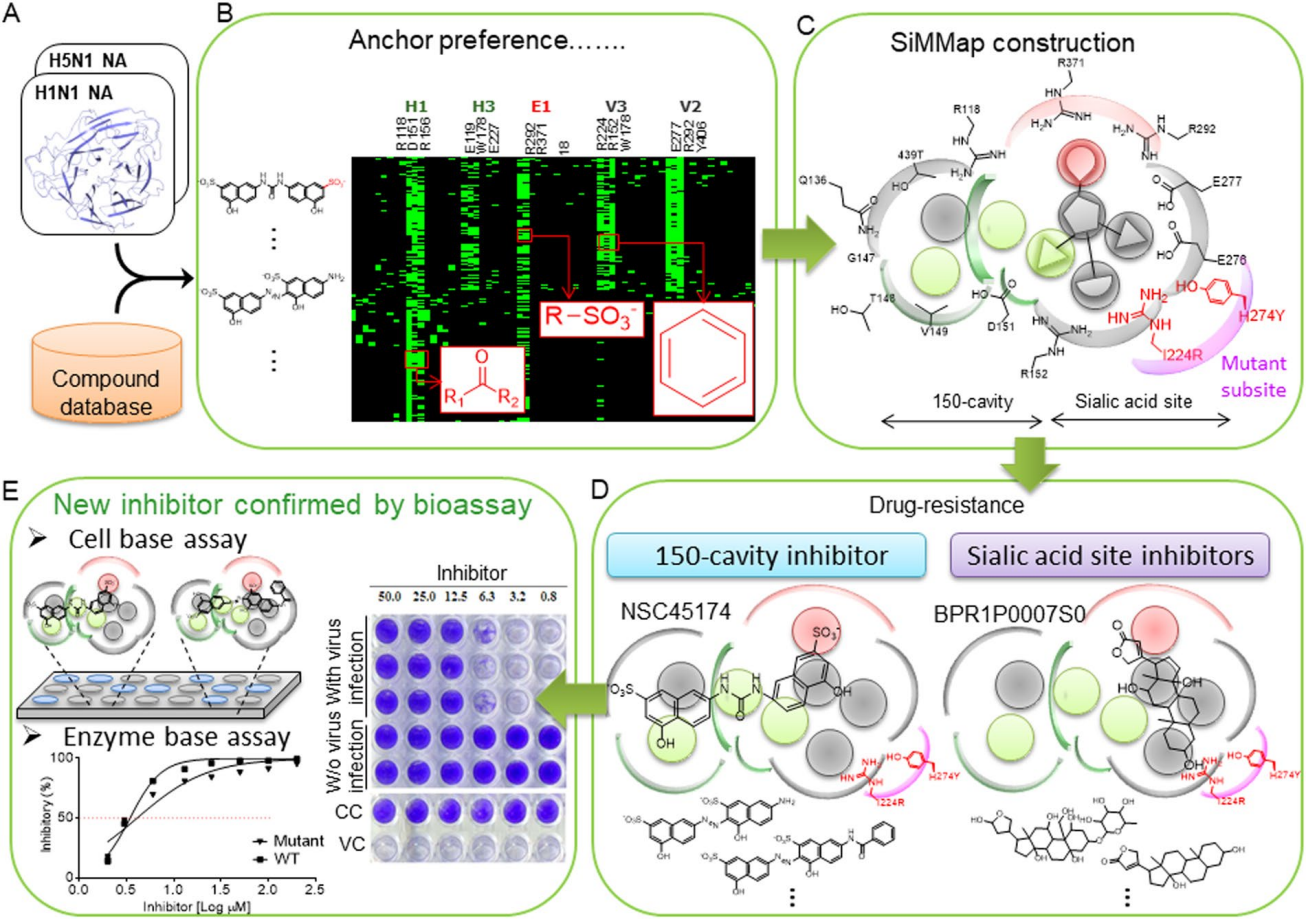
Framework of study. (**A**) Virtual screening for NA inhibitors by docking compounds from NCI database and in-house compounds. (**B**) Consensus interactions between docked compounds and NA residues. (**C**) Site-moiety map for revealing the most favorable binding pockets. (**D**) Groups of potential inhibitors. Compounds are ranked according to their site-moiety map scores and further classified into two groups according their locations. (**E**) Validation of potential inhibitors through cellular and enzymatic assays.

Figure 1 presents the overview of creating SiMMap models for identifying novel NA inhibitors. First, we docked 265,242 compounds from the NCI database and 2,000 in-house compounds into binding sites of H1N1 and H5N1 NAs using the docking program iGEMDOCK^33^ (Fig. 1A). Based on the docking scores, 1,000 of the top-ranked compounds were selected to establish site-moiety maps of H1N1 and H5N1 NAs. Next, interaction profiles between the 1,000 compounds and NAs were generated using iGEMDOCK (Fig. 1B). To validate the docking tool, we docked the co-crystalized ligands into two N1 structures, H1N1 (PDB ID: 3B7E) and H5N1 (PDB ID: 2HU4), by using iGEMDOCK. The docking results shows that the co-crystalized ligands (green) and the docked poses (blue) are similar within the binding sites (Fig. S1). Consensus interactions between compound moieties and binding pockets were identified from the profiles and regarded as the anchors (Fig. 1C). An anchor consists of (1) a binding pocket with consensus interacting residues, (2) moiety preferences of the pocket, and (3) types of interactions (electrostatic (E), hydrogen-bonding (H), or van der Waals (V) interactions) between the pocket and the moieties^32^. These anchors form the site-moiety maps and represent physicochemical properties of H5N1 and H1N1 NAs. Afterwards, the docked compounds were divided into two groups according to their locations of docked poses (Fig. 1D). The group-1 compounds match the anchors in the 150-cavity and sialic acid sites, while the group-2 compounds only match the anchors in the sialic acid site. Group 1 compounds that form interactions with I224 or Y274 were removed. Finally, for each group, the docked compounds were re-ranked by their site-moiety map scores. Potential inhibitors were selected for bioassay based on their scores, availabilities, drug-like properties, and domain knowledge (Fig. 1E).

### Anchors in 150-cavity

**Figure 2:**
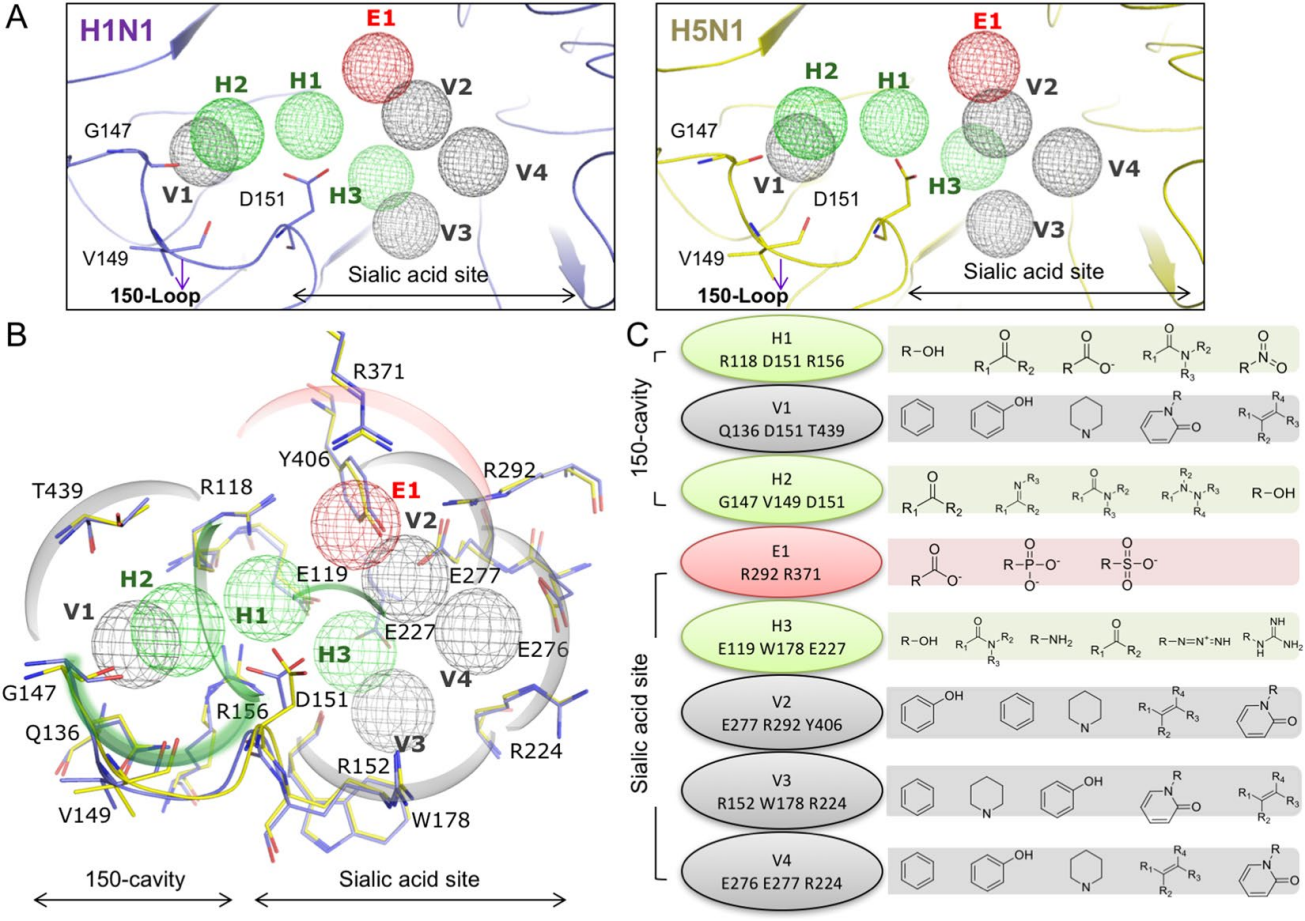
Site-moiety maps of NAs. (**A**) Anchor locations of H1N1 and H5N1 NAs. (**B**) Anchor residues. (**C**) Moiety preferences of anchors. H1, V1, and H2 are located in the 150-cavity, while the sialic acid binding site contained five anchors. Green, gray, and red anchors present the hydrogen, van der Waals, and electrostatic anchors, respectively. H1N1 and H5N1 NAs are colored by slate and yellow, respectively.

The SiMMaps, including eight anchors (E1, H1-H3, and V1-V4), of H1N1 and H5N1 are similar (Fig. 2A). Anchors H2 and V1 are located within the 150-cavity (Fig. 2B). The H1 anchor is located in the passage between the sialic acid binding site and the 150-cavity and contains three polar residues, R118, D151, and R156. The H1 anchor has preferences for hydroxyl, ketone, carboxylic acid, amide, and nitro group moieties (Fig. 2C). Most of these moieties form hydrogen bonds within this polar pocket. Therefore, compounds designed to occupy the 150-cavity may possess better affinities if they contain a polar moiety to form hydrogen bonds.

The H2 anchor is situated in the middle of the 150-loop (Fig. 2B). This anchor consists of residues G147, V149, and D151 and prefers to interact with polar moieties, which include ketone, imine, amide, hydrazine derivatives, and hydroxyl moieties (Fig. 2B). The residues Q136, D151, and T439 form the V1 anchor, which is located in the center of the 150-cavity (Fig. 2B). The V1 anchor also has a high preference to form interactions with bulky moieties that include aromatic rings, phenol, heterocyclic group, oxohetarene, and alkene moieties (Fig. 2C). The location and moiety preferences of H2 and V1 anchors suggest that an aromatic ring with polar moieties (*e.g*., aniline and phenol moieties) may be sterically and physicochemically complementary to the 150-cavity.

**Figure 3:**
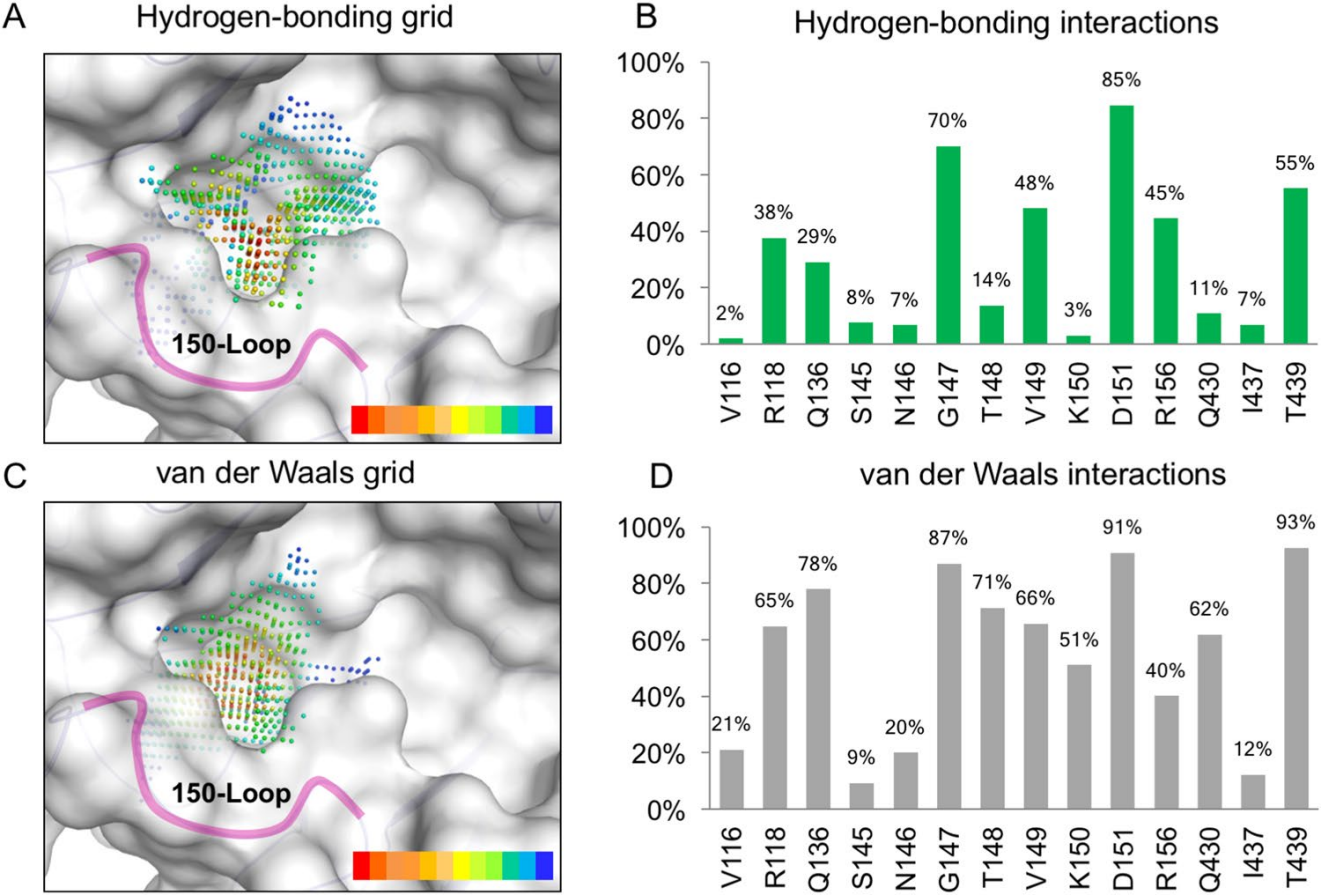
Interaction preferences of residues of the 150-cavity. Interacting atom distributions of compounds on the closed form of 150-cavity in terms of (**A**) hydrogen bonding and (**B**) van der Waals contacts. The interacting atoms are shown as grids within the binding site. Red dots represent spots occupied by highest number of different compounds, while blue dots represent the least in number. Interaction percentages of (**C**) hydrogen-bonding interactions and (**D**) van der Waals interactions for residues in binding site.

We further identified the residues that form hydrogen bonds within the 150-cavity. The interaction profiles between residues of the 150-cavity and the compounds were generated by iGEMDOCK^33^. The grid that overlays the 150-cavity is spaced 0.5 Å and represents the interaction frequency of the docked compounds (Fig. 3). This grid identified key hydrogen bonds and van der Waals interactions that occur within the 150-loop. We observed an increase in hydrogen bond interactions with the 150-loop, which consists of residues 147–152, in the open conformation. For the hydrogen-bonding grid, there was a high preference for 150-loop residues G147, T148 and D151 (Fig. 3A,B). This suggests that a large polar moiety can displace the water molecules within the network and lock the 150-loop into the open conformation (Fig. 3A). In contrast, residues 147–151 of the 150-loop showed a high degree of forming van der Waals interactions (Fig. 3C,D). Together, this suggests that new inhibitors can exploit the interactions to increase potency.

Crystal structures reveal that water molecules are often located at the H1 and H2 anchors in the closed and open conformations (Fig. S2). In each conformation, the water molecules form a hydrogen-bond network with the 150-loop residues, which may stabilize their conformation. The overlap of the 150-cavity reveals that the water molecules can change interactions between residues depending on the conformation (Fig. S2). Thus, the hydrogen-bond network is important in locking the NA in an open or closed confirmation. A compound with polar functional groups in the H1 and H2 anchors has greater potential to form such hydrogen-bond networks.

### Anchors in sialic acid binding site

The NA SiMMaps consist of five anchors (E1, H3, and V2-V4) in the sialic acid binding site (Fig. 2A). The NA subtypes shares conserved physical-chemical features for the sialic acid binding. The E1 anchor is a positively charged pocket that contains three highly conserved residues (R118, R292, and R371)^34^ that often form electrostatic or hydrogen-bonding interactions with negatively charged or polar moieties of the docked compounds. These residues are highly conserved in NAs of subtypes N1 and N2^24^. There is also a preference for interacting with the carboxylate moiety with this anchor. Apart from the carboxylate moiety, preference for sulfonic acid and phosphonic acid moieties were also identified from the docked compounds (Fig. 2C). The H3 anchor possessed a polar binding pocket with residues (E119, W178, and E227) that prefer polar moieties (*e.g*., hydroxyl group, carboxylic amide, ketone, and amine). In addition, sialic acid, zanamivir and oseltamivir consistently form hydrogen bonds with the anchor residues of the H3 anchor^10,25,35^. Furthermore, E119G mutation will reduce zanamivir susceptibility 1,400-fold^36^, suggesting this anchor could play an important role in designing NA inhibitors for the sialic acid binding site.

Three residues (E277, R292, and Y406) form the V2 anchor (Fig. 2B). Y406 is the catalytic residue for cleavage of the substrate^37^ and mutations with Y406 can have a drastic effect on NA activity^38^. Subsequently, the V3 anchor prefers hydrophobic moieties (e.g., aromatic ring, heterocyclic group, alkenes, phenol, and oxohetarene) and contained R152, W178, and R224 residues (Fig. 2B). Crystal structures (PDB codes 3B7E^25^, 2HU4^10^, and 1MWE^35^) reveal that acetamido moieties of sialic acid, zanamivir, and oseltamivir form interactions with W178 in this anchor.

Finally, the side chains of anchor residues R224, E276, and E277 in V4 anchor often form van der Waals with hydrophobic moieties of the docked compounds. The van der Waals interactions in the hydrophobic pocket are required for the binding process of oseltamivir^39^. However, the H274Y mutation produces a strain resistant to oseltamivir^39,40^. As a result, this mutation can alter the hydrophobic pocket to reduce the binding affinity of oseltamivir^41^. In addition, the dual mutation (H274Y and I222R) causes ~20, ~12,000, and ~7,500-fold reduction in NA inhibition for zanamivir, oseltamivir, and peramivir, respectively^19,26^. These mutations may limit the necessary rotation of V4 anchor residues to accommodate inhibitors and reduce their potency^42^. Based on the above analysis and results, it is concluded that the anchors of NA often play important roles in catalysis or substrate binding and the moiety preferences can be useful for drug design.

To further understand relationships between the anchors and NA inhibitors, we collected and analyzed known inhibitors including GS4071, zanamivir, zanamivir analogues from structure-activity relationship studies, and ATA since these compounds contain various moieties in the anchors of NA (Fig. S3)^43–48^. The moiety compositions between these compounds and the SiMMap anchors provide clues for lead optimization. For example, the compound 3a, an analogue of GS4071, has a phosphonate substitution at the E1 anchor that slightly enhances the IC_50_ value from 1 nM to 0.3 nM. Another example is the carbocyclic analogue 53, which contains an aromatic moiety to increase the inhibitory activity. The above results reveal that the anchors of the sialic acid binding site are often involved in catalytic process or are essential to the binding of substrate and inhibitors (e.g., zanamivir and oseltamivir). However, there are already reports of drug-resistant mutations for these drugs^17,49^. Most of these mutations are involved with residues within the anchors sites of the sialic acid. This includes the mutations R292K (E1 and V2) and E119V (H1) to name a few^16,42^.

### Identified novel inhibitors

**Figure 4:**
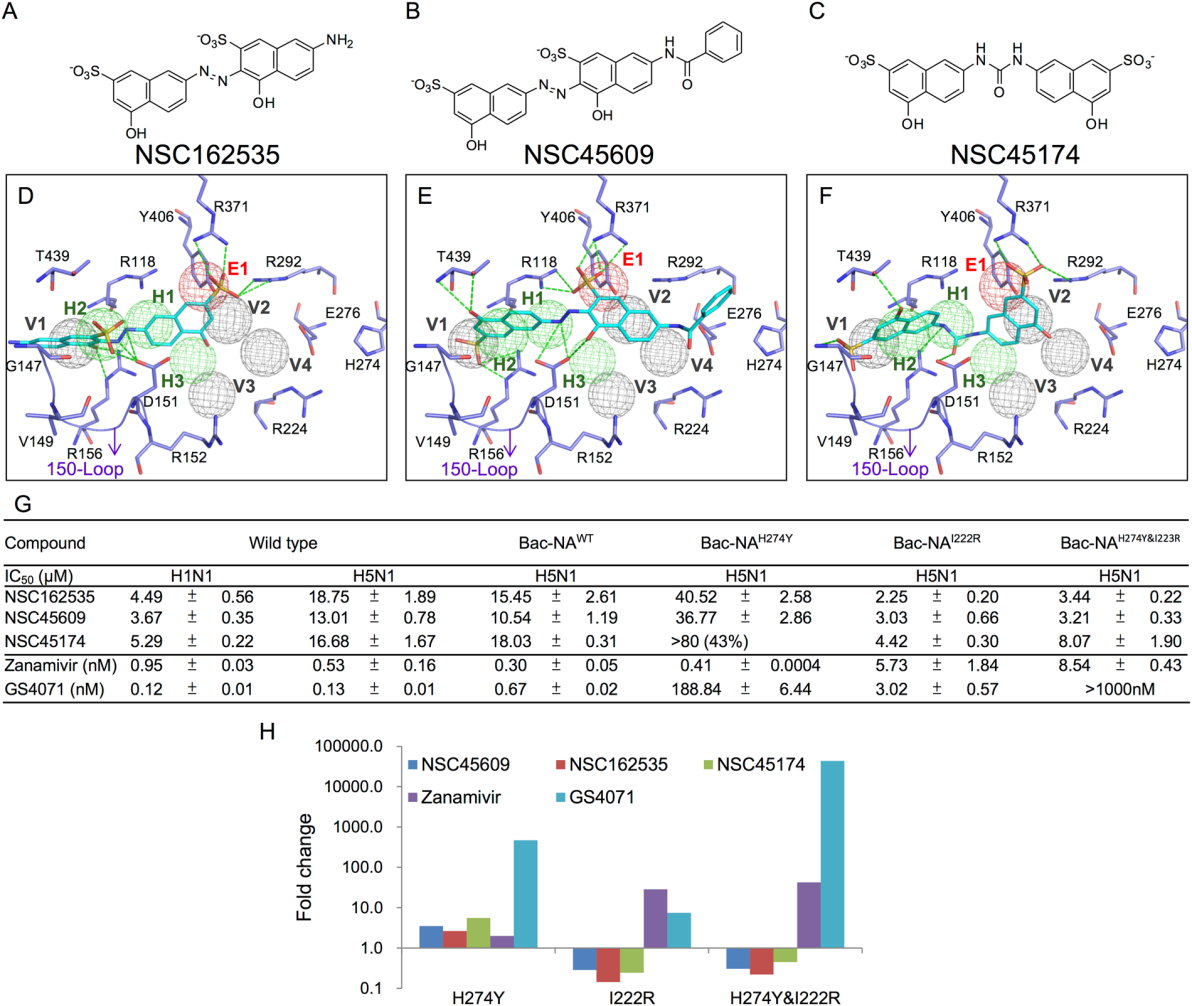
Identified NA inhibitors. (**A**–**C**) 2D structures of identified compounds. (**D–F**) Docked conformations of the inhibitors in H1N1 (residues in purple). (**G**) Enzyme-based assays of the inhibitors. Experiments performed in triplicate. Standard deviation is listed as shown. (**H**) The fold change in IC_50_ values of the inhibitors for H274Y, I222R, and H274 and I222R mutant strains. The green dashes represent hydrogen-bonding interactions. Anchors and residues are listed as shown.

Based on the SiMMap scores, we obtained 24 top-ranked compounds that have interactions with residues within the 150-cavity. Of these 24 compounds, three new inhibitors, named NSC45609, NSC45174, and NSC162535 with low IC_50_ values for H1N1 (<10.0 μM) and H5N1 (<20.0 μM) NAs were identified (Fig. 4). Furthermore, these three inhibitors formed interactions in five, four and five anchors, respectively (Fig. 4D–F).

The three new 150-cavity inhibitors contained sulfonic acid moieties and aromatic moieties. The former moieties form electrostatic interactions with R118, R292, and R371 of the E1 anchor, and the latter moieties make van der Waals contacts with Q136, D151, and T439 of the V1 anchor (Fig. 4D–F). The sulfonic acid moiety is a negatively charged moiety, which is similar to the carboxyl groups found on zanamivir, oseltamivir, and the substrate sialic acid. Although the urea moiety of NSC45174 contains an azo moiety, hydrogen-bonding interactions were still formed within the H2 anchor. Importantly, the anchor H2 contains residues V149 and D151, which showed high probable hydrogen-bond interactions (Fig. 3B). Similarly, the sulfonic acid moiety of NSC162535, and the hydroxyl moieties of NSC45609 and NSC45174 yielded consistent hydrogen bonds with the H2 anchor. It should be noted that these three new 150-cavity inhibitors were not located within the V4 anchor, which contains residues I222 and H274, suggesting that these inhibitors may not be affected by the two drug-resistant mutations R292K, N294S and H274Y^42^.

All three of the identified inhibitors contain a sulfonic acid and hydroxyl moiety located on the left naphthalene ring. This allows the inhibitors to form hydrogen bonds with amino acid residues within the 150-cavity (Fig. 4D–F). In contrast, analogous compounds, identified as NSC148367 and NSC47716, do not contain these groups in the same location (Fig. S4). Compound NSC148367 does not bind within the sialic acid site (Fig. S4A). While parts of the compound can be located within the 150-cavity, it does not create sufficient interactions with the H1 anchor. Likewise, compound NSC47716 does not have a large polar moiety to properly interact with the anchor sites (Fig. S4B). The other analogous do not properly dock within the active site. The identified inhibitors, when compared to these analogous compounds, create sufficient interactions within the 150-cavity to inhibit NA function. Thus, slight modifications to increase 150-cavity specificity to non-inhibiting compounds my yield new and effective NA inhibitors.

We subsequently tested the inhibitory activities of the 150-cavity inhibitors on H1N1 and H5N1 wild-type and with H274Y (NA^H274Y^), I222R (NA^I222R^), and H274Y & I222R (NA^H274Y&I222R^) mutant strains. For the H274Y mutation, NSC162535 and NSC45609 contained showed better inhibition than GS4071 (oseltamivir carboxylate), though zanamivir continued to show a greater inhibition for this strain (Fig. 4G). The identified compounds showed comparable inhibition for the I222R mutant strains. Finally, the identified compounds showed less than 10 µM inhibition for the H274Y and I222R mutant strains (Fig. 4G). The fluorescent fold change of the identified compounds performed better for the I22R and the H274Y/I222R strains compared to zanamivir and GS4071 (Fig. 4H).

**Figure 5:**
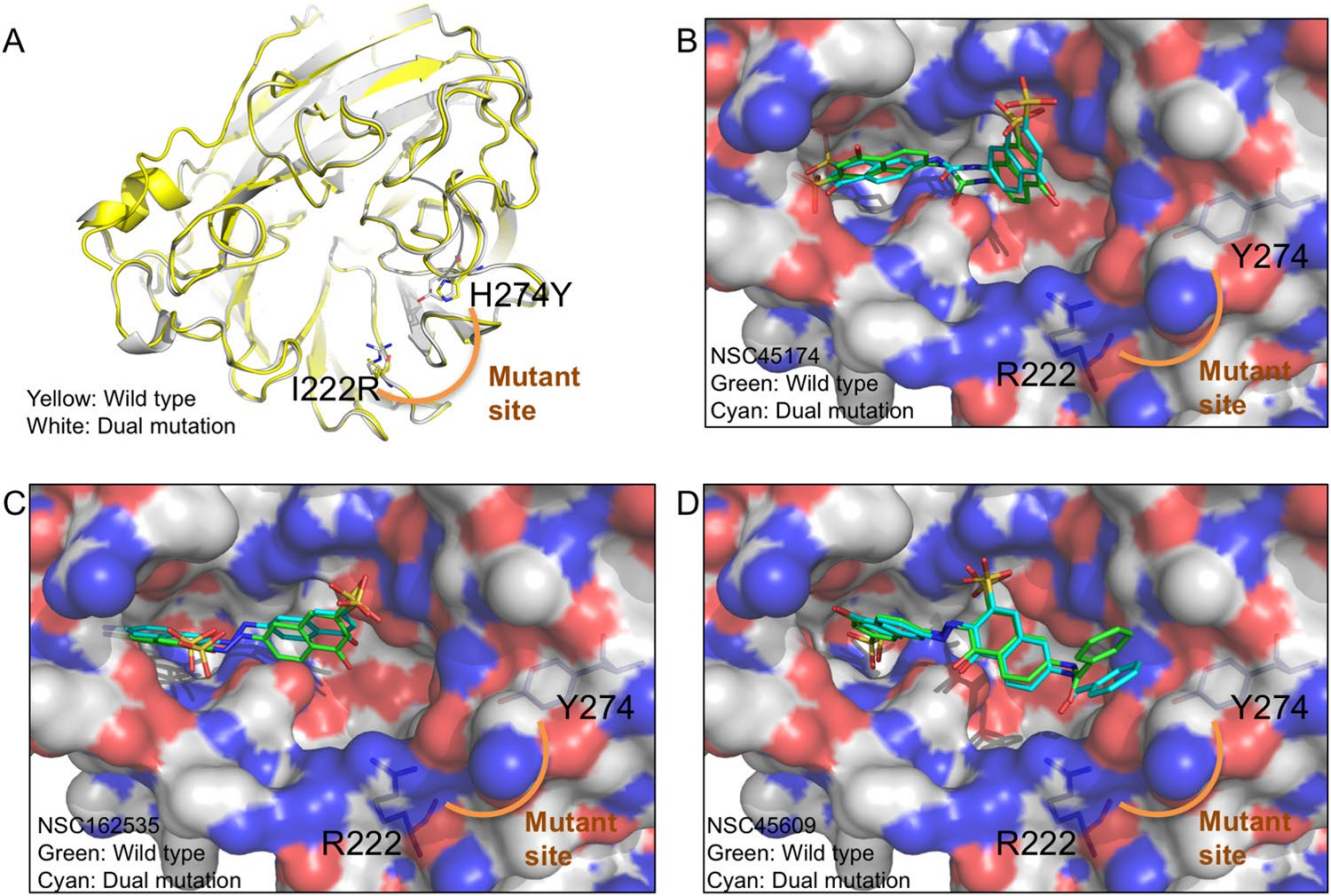
Identified compounds do not form interactions with mutant residues. (**A**) The cartoon model for a wild type and dual mutation of H5N1 (PDB ID: 2HTY) are superimposed. Mutant residues are labeled as shown. The identified compounds (**B**) NSC45174, (**C**) NSC162535, and (**D**) NSC45609 were docked into both wild type and the MDR NA with their poses superimposed. The compounds show similar binding conformation and do not form interactions with the mutant site.

We next compared the structure of wild type and MDR NAs. The structure of MDR NA was derived using a homology modeling approach^50^. The protein sequence of the strain NIBRG14 (H5N1) with the H274Y/I222R mutations was submitted for the server. The structure of H5N1 was then used as the template to obtain the structure. Besides the H274Y/I222R mutant sites, the dual mutant and wild type structure do not significantly differ (Fig. 5A). However, the surface view of the dual mutant structure shows a change in volume and polarity within the binding site due to the H274Y/I222R residue mutation^51^. The I222R mutation has been previously shown to reduce the volume of the binding site^51^. The substitution of tyrosine for the H274Y mutant may further reduce the size of the hydrophobic pocket of the binding site^29^. When the docking poses of the inhibitors from both wild-type and mutant structures were superimposed, we did not observe a significant difference in their position within the binding cavity (Fig. 5B–D). Furthermore, the three compounds do not form interactions with the mutant sites and do not contain steric hindrance.

**Figure 6:**
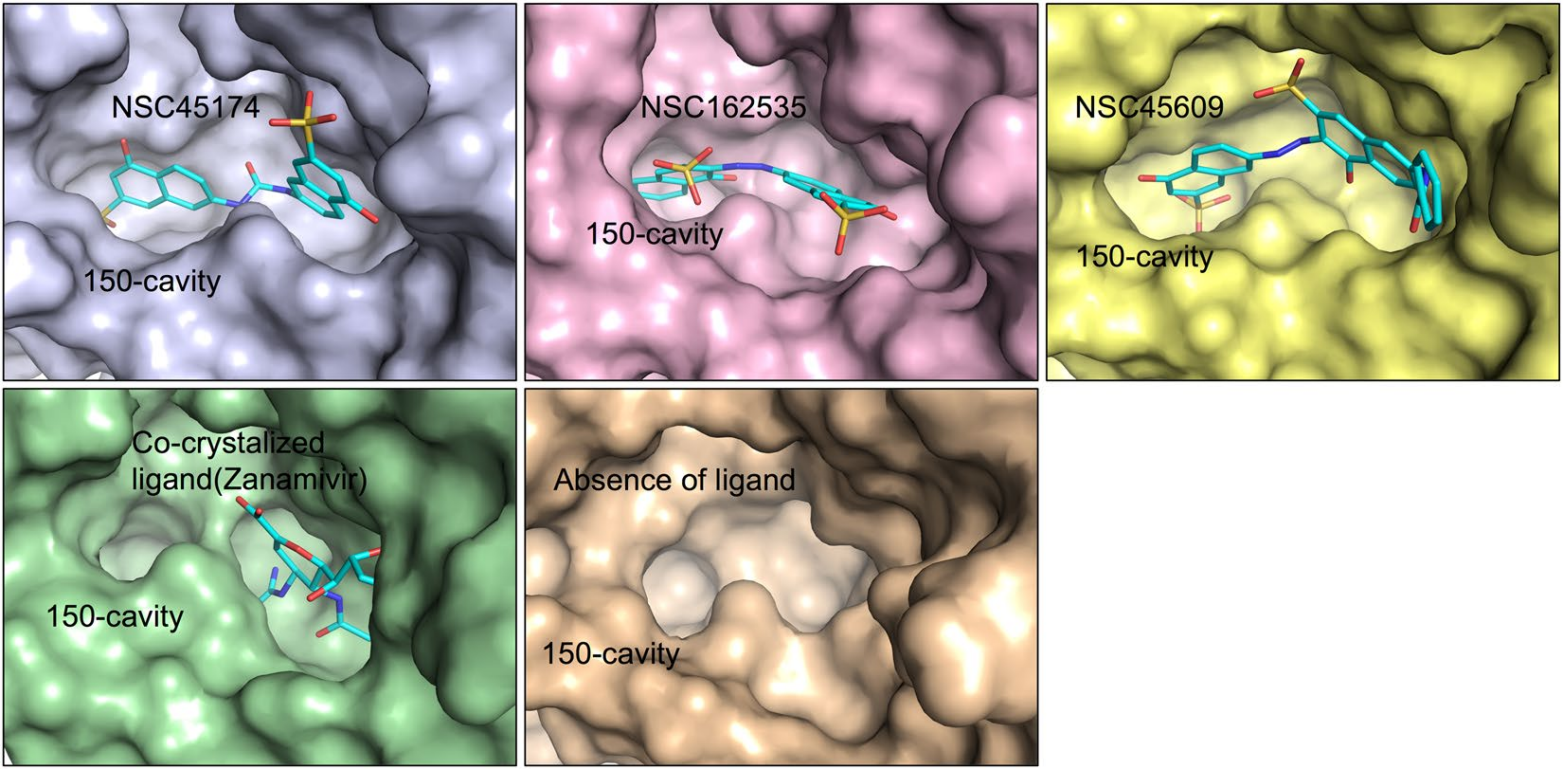
MD simulation of identified compounds lock the 150-cavity in the open conformation. The MD simulation for the open conformation of NA was performed with compounds NSC45174, NSC162535, NSC45609, co-crystallized ligand (zanamivir), and in absence of a ligand for 10,000 picoseconds. The stable structure for each condition was rendered as a surface model. The identified compounds lock the NA structure in the open conformation to produce the 150-cavity. In contrast, the co-crystallized ligand lacks the 150-cavity. A MD simulation of open conformation of NA in absence of ligands produced an open 150-cavity. The 150-cavity is listed as shown.

To explore the open/close conformational changes in presence or absence of the ligands, we performed a molecular dynamic (MD) simulation using the NA structure (PDB ID: 2HTY) in the presence or absence of the ligands for 10,000 picoseconds (Fig. 6). The MD simulations of protein and protein-ligand systems resulted in stable final structures as observed by the lower energy forms observed after 6 ns in all simulations (Fig. S5). At the end of the simulation, the three identified compounds locked the 150-loop and produced an open cavity conformation (Fig. 6). In contrast, the co-crystallized ligand, zanamivir, induced a closed cavity conformation (Fig. 6). In addition, when we simulated an open form structure without a ligand, an open 150-loop cavity conformation was produced (Fig. 6). These experimental results support that these compounds can bind to the-150 cavity to form interactions with the cavity residues.

**Figure 7:**
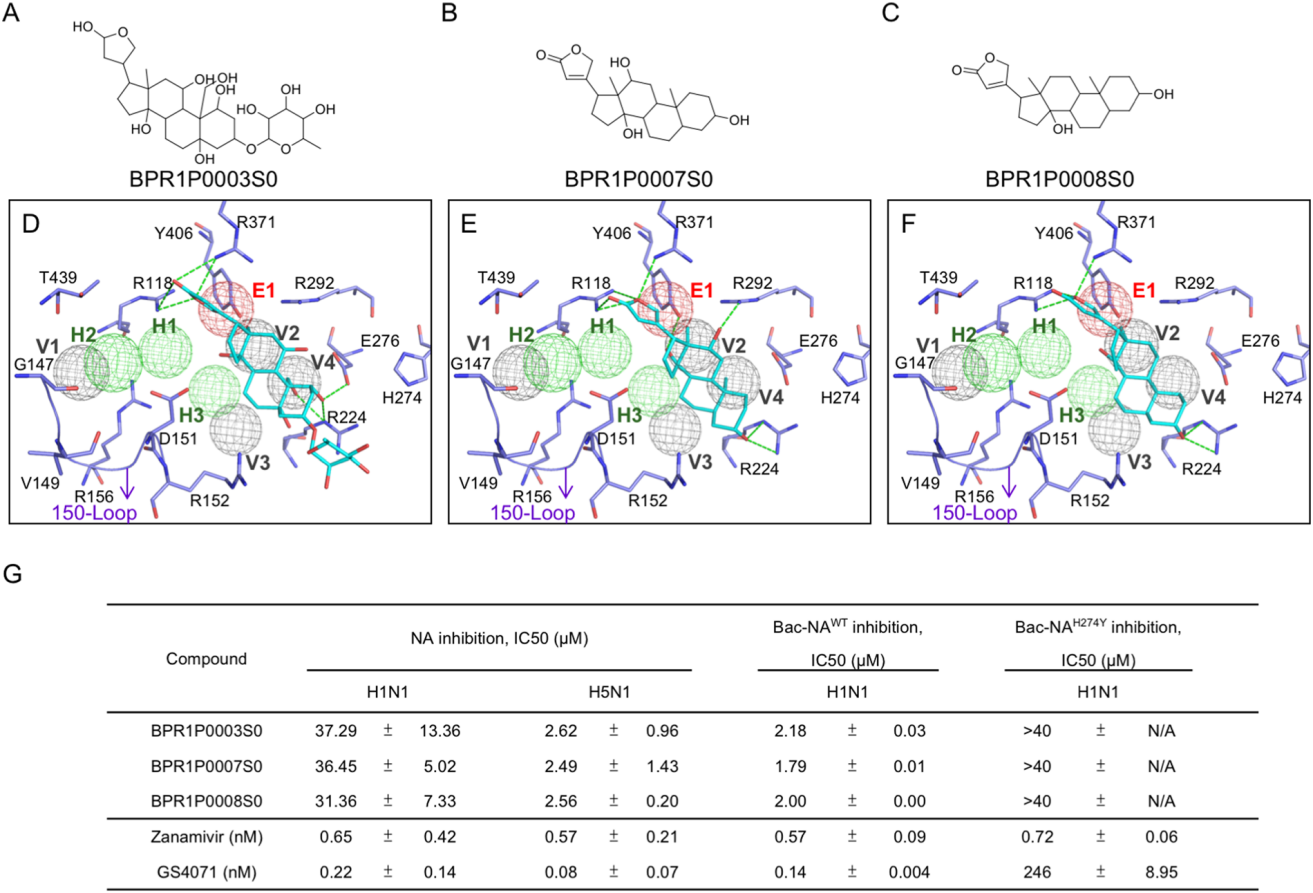
Identified inhibitors located in the sialic acid-binding site. (**A–C**) Compound structures of identified inhibitors. (**D–F**) Docked conformations of the inhibitors in the binding site of H1N1 (residues in purple). (**G**) Enzyme-based assays of the inhibitors. The green dashes represent hydrogen-bonding interactions. Experiments performed in triplicate. Standard deviation is listed as shown.

Our screening process also identified three sialic-acid site inhibitors, BRP1P0003S0, BPR1P0007S0 and BPR1P0008S0, from our in-house compounds. These compounds consistently matched anchors E1, V2 and V4 (Fig. 7A–F). These three inhibitors included 9a,11a‐dimethyl‐hexadecahydro‐1 H‐cyclopenta[a]phenanthrene‐3a,7‐diol moiety, which yields stable van der Waals interactions with the residues R224, E276, E277, and R292 of the V2 and V4 anchors. The moiety also forms a hydrogen bond with R224. 2-furanone moieties on inhibitors BPR1P0007S0 and BPR1P0008S0 yielded several hydrogen bonds with residues R118, R371, and Y406 of anchor E1. In addition, there are van der Waals interactions formed between the two compounds and the residue I222. Only BPR1P0007S0 makes one additional hydrogen bond with R292, while BPR1P0003S0 makes one additional hydrogen bond with E276. Overall, the hydrogen bonds of these inhibitors appear mostly identical. Furthermore, the tetradecahydrophenanthrene moieties of these inhibitors form van der Waals interactions with residues R371 and R152.

One of the cyclohexane rings of the tetradecahydrophenanthrene moiety forms van der Waals interactions with the sidechain of R152 of anchor V3. As for anchor V4, the sidechains of R224 and E276 form a fitting pocket for one of the terminal cyclohexane rings, while residue R292 of the E1 anchor is in smooth alignment with the other terminal cyclohexane ring at the other end. However, these sialic inhibitors bind in areas prone to have mutations. For instance, the H274Y mutation occurs in the sialic site and will also reduce the interaction energy between residues R224 and E276. These contact points are important in the inhibition process of NA^52^. In addition, inhibitory activities of these inhibitors were tested on H1N1 and H5N1 NAs with H274Y (NA^H274Y^). NA inhibition assays revealed the compounds having high potency with wild-type strains. Meanwhile, H5N1 NAs with H274Y (NA^H274Y^) mutation did not show a strong inhibitory effect (Fig. 7G). Thus, the three compounds show inhibitory effect to wild-type NAs, but weak effect towards strains with mutations in the sialic acid site. This is due to the binding to sialic-acid site, where higher frequency of mutations occurs^17,53^.

We further compared these six new inhibitors with known NA inhibitors. The NA inhibitors were collected from BindingDB^54^. Atom-pair fingerprint of each compound was generated using RDKit Fingerprint in KNIME^55^. The new inhibitors and known NA inhibitors that interact with the NA binding site were clustered using hierarchal clustering approach. Pearson’s correlation coefficient was used as the distance measure to compare fingerprint similarity between two compounds. A heat map representing the similarity matrix was generated and showed that the identified compounds had little resemblance with other structures (Fig. S6). This result suggests that the new inhibitors contained novel structures.

### Testing effects of new inhibitors using cell-based experiments

**Figure 8:**
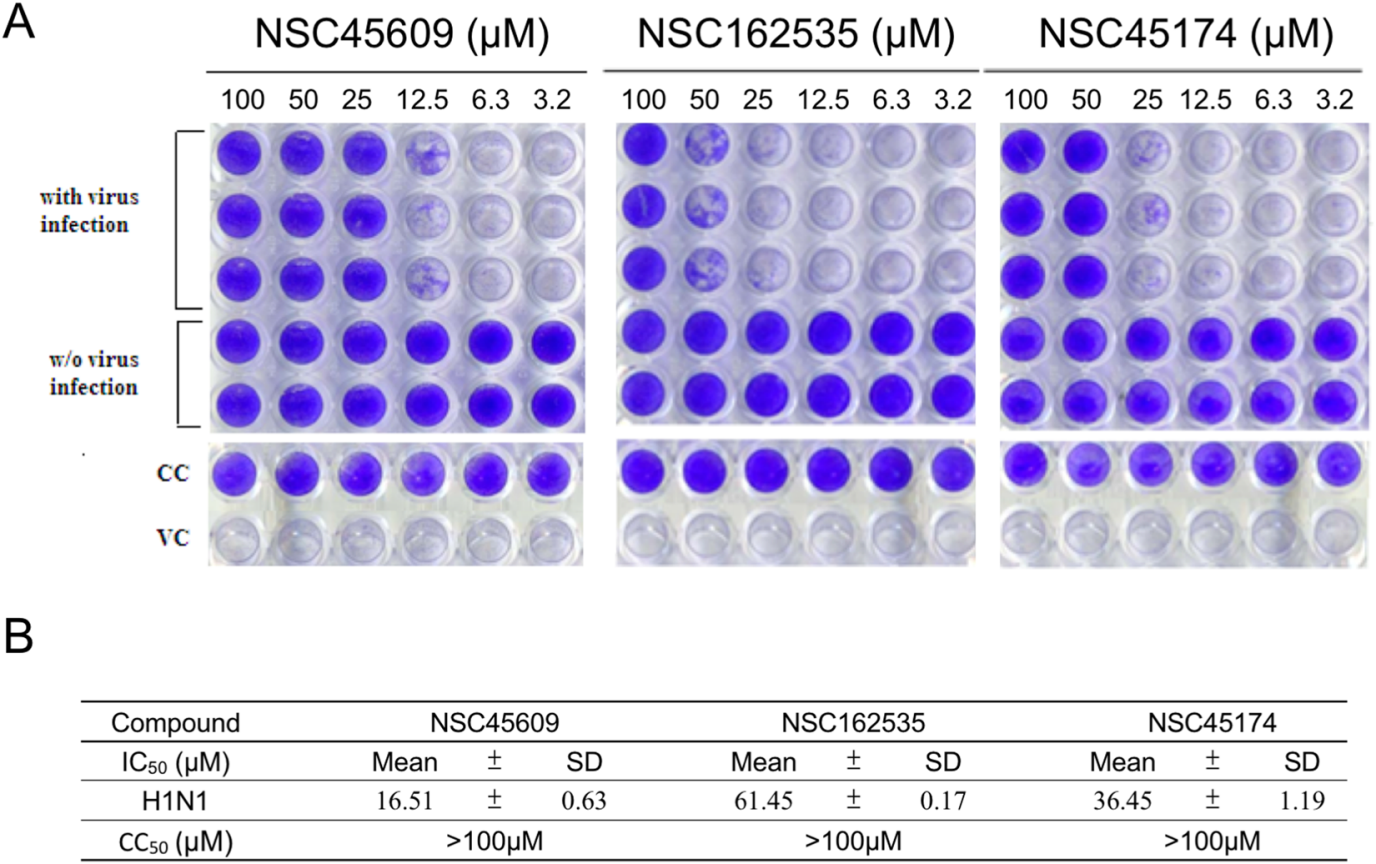
Effects of influenza virus in cytopathic effect inhibition assays of novel inhibitors. (**A**) Infection of MDCK cultured cells with influenza viruses was conducted in the presence of different inhibitor concentrations. The cells are stained with crystal violet after incubation. The presence of the blue color indicates living cells without the viral infections. Absence of blue color indicates the infecting virus causes lysis of the host cell. Virus control (VC) and cell control (CC) are listed as shown. Numbers above each well represents concentration of inhibitor used in µM. (**B**) The mean IC_50_ values of the inhibitors against H1N1 virus and the stare listed as shown. Experiments performed in triplicates with the standard of deviation for each mean listed in table.

Because of our interests in discovering 150-cavity inhibitors, which is less likely to interact with mutations found in the sialic acid sites, we focused on the three inhibitors identified from the NCI database. The inhibitions of these three new inhibitors were tested using a cytopathic effect assay. In the assay, MDCK cells were lysed 64 hr after viral infection, as shown in the virus control (VC) column. Inhibitors were added to the virus-infected cells by two-fold serial dilution starting with a concentration of 100 µM (left most column). As shown in Fig. 8A, the results indicate that influenza viruses cause CPE on infected cells after 64 hr post infection (p.i). In contrast, in the virus control (VC) column, wells without viral infections are colored blue as an indication of the presence of living cells. NSC45609, NSC162535 and NSC45174 were effective in abrogating influenza virus infection in a dose-dependent manner, as evidenced by the gradient of blue color observed across the columns of wells with compound treatment. NSC45609 showed consistent blue color at 25 µM to 100 µM, whereas NSC162535 requires a minimum of 50 μΜ to reach such consistency in color. NSC45174 is in between the two previously mentioned inhibitors, landing at 50 to 100 μM in concentration. We also monitored the cell viability and general toxicity of inhibitors as cell control (CC) on replicate plates. These compounds showed a CC_50_ value of more than 100 μΜ, while maintaining low toxicity in MDCK cells. The compounds inhibited >50% of the cytopathic effect without causing apparent cytotoxicity for H1N1 infection (Fig. 8). The concentration required for the identified compounds to reduce the CPE of the virus by 50% (IC_50_) for NSC45609, NSC162535, and NSC45174 were 16.5, 61.5, and 36.5 μM, respectively, for H1N1 induced CPE (Fig. 8B). In the CPE assay, GS4071 (oseltamivir carboxylate) was used as the positive control, the IC_50_ of GS4071 obtained is 0.02 ± 0.0031 μM in different experiments (data not shown).

The above results suggest that inhibitor NSC45609 as the most potent, with NSC45174 and NSC162535 ranked second and third, respectively. These results indicate that the three inhibitors are strong lead compounds for designing new inhibitors to overcome drug resistance introduced by mutations within the sialic site for N1 NAs without causing significant cytotoxicity.

## Discussion

Our study discerned the structural characterization of the 150-cavity of NA to identify novel inhibitors that may overcome influenza viruses with resistance to conventional drugs. While group-1 NA consists of N1, N4, N5 and N8, the virtual screening in this study focused on N1. This is due to the emergency of multidrug resistance in H1N1 and H5N1 viruses^56^ and the recent outbreaks of avian influenza H5N1^1–3^. The establishment of SiMMap models provided an efficient method of predicting the interactions between new compounds and NAs. We identified anchors, which led to a deeper understanding of the interaction modes of this protein, thus signifying the importance of these binding environments. For example, in the models, the determination of the E1 anchor region within the sialic acid site allowed us to determine particular traits of the neighboring residues, which in turn assisted us in the listing of the carboxylate, sulfonic and phosphoric moiety preferences of this particular anchor. Furthermore, these models also make it possible to screen for compounds with novel scaffolds, avoiding common mutations that may arise with NAs. Information regarding interaction preferences of the residues within the 150-cavity provides us with more details of the most important amino acid sidechains. For instance, the 150-cavity, which is made up of residues 147–152, has been thought of as a new site for some inhibitors^24,53^. Likewise, the inhibitors identified in our study showed a very high preference for the 150-cavity. Together, the SiMMap models, including the moieties, residues, and binding anchors within, provide clues for identifying novel inhibitors.

The mutations that occur with the residues around the sialic site decrease traditional NA inhibitors efficacy, making the 150-cavity a valued site for new inhibitors. Mutations with residues located in the sialic acid site, such as I222R and H274Y, have been shown to reduce the size of the hydrophobic pocket, thereby increasing its oseltamivir resistance^29,39,40,51,57^. The reduction in size of the hydrophobic pocket also sterically clashes with inhibitor interactions. A past study suggested that NA mutations outside of the active site may play a role in oseltamivir resistance^27^. Nevertheless, the three identified inhibitors in this study show preference for binding within the 150-loop (Fig. 4). MD simulation revealed that the identified inhibitors was not greatly affected by the I222R/H274Y dual mutations. We compared the structures of wild-type and multiple drug resistant NAs and found differences in volume and polarity within the active site (Fig. 5). Therefore, inhibitors that can exploit the 150-cavity or force the NA into an open conformation presents a favorable method of inhibiting NA activity.

The new inhibitor NSC162535 possesses an interacting sulfonic acid moiety that distinguishes itself from the rest of the others. This particular moiety forms hydrogen bonds with residues of the H2 anchor region, also within the 150-cavity. In fact, this moiety is found on inhibitors NSC45609 and NSC45174 and forms interactions with anchor V1 within the 150-cavity (Fig. 4B–C). The identified inhibitors contain at least four benzene rings, grouped in twos, and joined together by an azo or urea moiety. In contrast, compounds NSC148367 and NSC47716, which are analogues of NSC162535, do not contain a hydroxyl group attached to the left of the benzene ring (Fig. S4). The absence of the sulfonic moiety greatly reduces the compounds’ ability to sufficiently inhibit NA. Therefore, apart from identifying new inhibitors, we also identified the major differences from non-inhibiting compounds that could yield slight modifications to compounds in the hope of creating NA inhibitors.

The identified moiety preferences for the anchors within the 150-cavity site become the guidance for the lead optimization process, which is a means of modifying functional groups on already existing drugs, such as GS4071. The carboxylate moiety of GS4071 can be replaced by a phosphonic group, which was observed in our study to form strong interactions within the 150-cavity site. In addition, the phosphonic group also forms favorable interactions to the E1 anchor residues.

Although there are existing drugs for inhibiting NA, many patients become resistant to treatment due to mutations in the neuraminidase enzyme. Mutations of residues in various anchor regions of NA, including E199V, D151E, H274Y, R292K, and N294S strains, may alter their characteristics and interaction modes, disrupting drug binding^53,58,59^. Our analysis of NSC45609, unlike the other two inhibitors, does not hydrogen bond with R292 in the E1 anchor region, but forms hydrogen bonds with other residues within the 150-cavity. Thus, identifying and creating compounds to exploit the 150-cavity may give an advantage for compounds to overcome the traditional mutant NA strains.

Analysis of the 150-open form structures indicates that several water atoms occupy the H2 anchor, which can encompass parts of the 150-loop (Fig. S2). Compounds that interact with the 150-loop may force the NA in an open conformation, which has been used as a structural target for designing new N1 inhibitors^60^. There exists a hydrogen-bonding network within the active site (Fig. 3). Strong hydrogen bonds can form within the 150-loop. Furthermore, the hydrogen bonding network changes according to the open or closed conformation (Fig. S2). Large polar moieties may displace water located within the bonding network observed. This occurs in the newly identified inhibitors. The sulfonic moiety occupies anchors V1 and H2, which contain 150-loop residues. The inhibitors’ affinity for the 150-loop further circumvents possible mutations within the sialic site^39,40,59^ and can lock the protein into the open-form. This is in contrast to the identified in-house compounds, which bind within the sialic site.

## Conclusions

This study demonstrated the utility and feasibility of a site-moiety map as a screening strategy for identifying novel inhibitors and optimizing lead compounds. Three new inhibitors were identified that occupied the 150-cavity. Furthermore, the identified inhibitors showed inhibition for oseltamivir-resistant NA strains containing with H274Y and I222R mutations. We further identified compound analogues that do not have the same inhibitory effect. Based on the site-moiety map, we found that the analogues miss the functional moieties that form hydrogen bond interactions within the 150-cavity site. These results reveal the advantages of the computational screening strategy to find new types of inhibitors for combating drug-resistant strains. Based on the experimental results, we believe that this screening strategy is advantageous to identify and design a novel class of inhibitors that may overcome the drug resistance.

## Methods and Materials

### Dataset preparation

To identify new inhibitors that could bind the 150-cavity, we selected 150-open form structures of NAs including H1N1 (PDB code 3BEQ^25^) and H5N1 (PDB code 2HTY^10^) for constructing site-moiety maps. These two structures were selected based on two criteria: resolution and conformation of the 150-loop if they were in open conformation. Compared to other PDB structures, the selected structures have the best resolution and open-form conformation. For the 150-open form of H1N1 NA, the binding site comprising the 150-cavity and the sialic acid binding site was defined to include the residues within a 10 Å radius sphere centered around the 150-loop (residues 147–152^5^) and zanamivir by superimposing a crystal structure of H1N1 NA (PDB code 3B7E^25^). This binding site was applied to derive binding sites of H5N1 NA using a structural alignment tool^61^.

The compounds used for virtual screening were collected from two compound libraries including NCI library and 2,000 in-house compounds. The structures of these compounds were obtained by CORINA^62^. The compounds were removed if their molecular weights are <200 or >650 Dalton.

The NA structure with I222R and H274Y dual-point mutations was derived using a homology-modeling server^50^. The protein sequence submitted for the server was from the strain NIBRG14 (H5N1) with the dual-point mutation, which was used to generate MDR NA. The unbound NA structure (i.e. 2HTY) was selected as the structure template. The binding site of the mutant structure was generated using the procedure described above.

### Virtual screening of compounds and construction of the site-moiety map

The compounds were docked into the binding sites of H1N1 and H5N1 NAs using iGEMDOCK^33^. The compounds were then ranked based on their docking scores. The 1,000 top-ranked compounds and their corresponding binding sites were submitted to the SiMMap server^32^. The server applied the iGEMDOCK program to generate protein-compound interaction profiles (Fig. S7A,B). iGEMDOCK considers three types of interactions, including electrostatic, hydrogen-bonding, or van der Waals interactions. Based on these profiles, consensus interactions between compound moieties and binding pockets consisted of conserved interacting residues were identified (Fig. S7C). In addition, moiety preferences of the binding pockets were identified from the top-ranked 1,000 compounds. The binding pockets, the moiety preferences of the pockets and pocket-moiety interaction type constitute the anchors of the SiMMaps (Fig. S7D).

### Identification of potential inhibitors

The anchors of the site-moiety maps can be divided into 150-cavity group and the sialic acid group based on their locations. The anchors (H1, H2, and V1) of the 150-cavity group were then used to identify potential inhibitors against mutant NA strains. For each compound, we generated a site-moiety map score using the SiMMap model^32^. Based on the scores, the compounds of NCI database and in-house library were re-ranked. The top-ranked compounds were further divided into two groups. Group 1 compounds match the anchors (*i.e*., H1, H2, and V1) in the 150-caivty and were filtered if they interact with the mutant residues. Group 2 compounds only match the anchors in the sialic acid site. Finally, potential inhibitors were selected for further testing based on their ranking, availabilities, drug-like properties, and domain knowledge.

### Comparing compound structure

The structures of the new inhibitors were compared with other NA inhibitors. The NA inhibitors were obtained from BindingDB^54^. Atom-pair fingerprint of each compound was generated using RDKit Fingerprint tool in KNIME^55^. A similarity matrix and hierarchal clustering using Pearson’s correlation coefficient was performed using the program morpheus (https://software.broadinstitute.org/morpheus/). Compounds were sorted based on similarity. Compounds with high similarity are colored red, while the compounds with low similarity are colored blue. Since the Food and Drug Administration approved drugs can set off PAINS alerts^63^, a PAINS filter was not applied to the virtual screening procedure to avoid the loss of any potentially interesting compounds^64^.

### Molecular Dynamic Simulation

Molecular dynamic simulations were performed in Discovery Studio^65^ following the Standard Dynamics Cascade protocol. The protein and protein-ligand systems were built by applying CHARMm^66^ force field and solvated by explicit periodic boundary conditions. The systems were first minimized using 1000 steps of steepest descent, 2000 steps of adopted basis NR, and heated to a target temperature of 300 K followed by equilibration, with a time step of 2 fs. Finally, the simulations were performed for a production time of 10,000 ps using default parameters, the non-bond higher cutoff distance set to 18, and the non-bond lower cutoff distance set to 16. The final conformations after completion of simulation were extracted and used for the analysis.

### Recombinant NA proteins preparation

The oseltamivir-resistant NA contains a H274Y single point mutation for strain H1N1, which was obtained as described as Hung *et al*.^45^. The oseltamivir-resistant NA with H274Y and I222R dual-point mutations for strain NIBRG14 (H5N1) was obtained as described as Hung *et al*. In brief, the NA expression constructs were co-transfected with linear BacPAK8 viral DNA into Sf9 insect cells as described previously. Recombinant baculoviruses were generated to express the wild-type, H274Y, H274Y and I222R mutants of NA originating from influenza N1 neuraminidase (NIBRG14 (H5N1))^45^. Total cell lysates were treated with pronase (2.5 mg/ml) for 1 hour at 22 °C to reduce unrelated cellular protein background noise and enhance specific signal.

### The Neuraminidase Inhibition Assay

The wild-type, H274Y, H274Y and I222R mutants of NA recombinant proteins are derived from baculovirus insect expression system^67^. The NA enzymatic activity was measured using the fluorogenic substrate MU-NANA as described^68^. Each assay contained the compound, the compound and substrate, or compound and 4-MU used as a control to eliminate false-positive signals. A serial dilution of inactivated virus stocks were pre-incubated with the test compounds for 30 min at 30 °C. The assay was conducted in 96-well plates containing active wild-type, H274Y, H274Y and I222R mutants of NA and 100 μM fluorogenic substrate per well in reaction buffer. The enzymatic reactions were then carried out for 1 h at 37 °C and then terminated by the stop solution. The fluorescence intensity of the product 4-MU was measured with Ex/Em 330/445 nm, respectively. The half maximal inhibitory concentration (IC_50_) for reducing different types of NA activity were then determined. The IC_50_ of GS4071 (oseltamivir carboxylate) was tested against NAs as a positive control in the NA inhibition assays.

### Cells and virus culture

Madin-Darby Canine Kidney MDCK cells (ATCC accession no. NBL-2) were grown in Dulbecco’s Modified Eagle medium (DMEM; Gibco) with 10% fetal bovine serum (FBS; Gibco), 100 U/mL of penicillin and streptomycin, and 2 mM L-glutamine. Influenza A virus (A/WSN/33) viruses were kindly provided by Dr. Shin-Ru Shih (Clinical Virology Laboratory of Chang Gung Memorial Hospital, Linkou, Taiwan). Virus amplification and titration was performed on MDCK cells.

### Inactivation of flu virus stock

To inactivate H1N1and H5N1viral infectivity, the cell culture suspensions of H1N1- and H5N1-infected MDCK cells were inactivated with 0.02% formaldehyde as described by Hung *et al*.^45^.

### Virus Induced-Cytopathic Effect (CPE) inhibition assay

To discover new types of inhibitors, we selected top-ranked compounds occupying the 150-cavity. In addition, we selected the modified zanamivir derivatives for bioassays. The bioassays, including virus induced-cytopathic effect (CPE) inhibition test and cytotoxicity assay, were used to evaluate the utility for identification and optimization of lead compounds. Madin-Darby Canine Kidney MDCK cells (ATCC accession no. NBL-2) were grown in Dulbecco’s Modified Eagle medium (DMEM; Gibco) with 10% fetal bovine serum (FBS; Gibco), 100 U/mL of penicillin and streptomycin, and 2 mM L-glutamine. Influenza A virus (A/WSN/33) viruses were kindly provided by Dr. Shin-Ru Shih (Clinical Virology Laboratory of Chang Gung Memorial Hospital, Linkou, Taiwan). Virus amplification and titration was performed on MDCK cells. The protocol of CPE inhibition assay has been described by Hung *et al*.^45^. The concentration of identified inhibitors to reduce the appearance of CPE in influenza virus-infected MDCK cells were measured by evaluating inhibition of virus induced cytopathic effect, 96 well tissue culture plates were seeded with 200 μL of 1 × 10^5^ cells/ml in DMEM with 10% FBS. Cells were incubated 18 to 24 h at 37 °C and then washed with PBS, and challenged with virus (moi 0.01) in 150 μL DMEM medium containing trypsin. After 1 hr viral adsorption, the infected cells were overlaid with 50 μL DMEM and various concentrations of identified inhibitors and incubated at 37 °C for 72 hr. At the end of the incubation, the cells were fixed with formaldehyde and stained with 0.1% crystal violet. The concentration required for the identified compound to reduce the CPE of the virus by 50% (IC_50_) was determined. Cell toxicity of the identified inhibitors were determined to detect general toxicity based on cell viability. The optical density was measured at OD590 nm in an ELISA reader.

## Electronic supplementary material

Supplementary Data
