# Supplementary material for: Identification of neuraminidase inhibitors against dual H274Y/I222R mutant strains
Source: Sci Rep. 2017 Sep 26;7:12336. doi: 10.1038/s41598-017-12101-3 (PMC5615050; doi:10.1038/s41598-017-12101-3)
Supplement: Supplementary file 1 — Supplementary Data [file 41598_2017_12101_MOESM1_ESM.pdf]

Identification of neuraminidase inhibitors against dual H274Y/I222R mutant strains

Kai-Cheng Hsu[1◎], Hui-Chen Hung[2◎], Wei-Chun HuangFu[1], Tzu-Ying Sung[3], Tony Eight Lin[1], Ming-Yu Fang[2], I-Jung Chen[2], Nikhil Pathak[4], John T.-A. Hsu[2,5*], Jinn-Moon Yang[3, 5*]

[1] Graduate Institute of Cancer Biology and Drug Discovery, College of Medical Science and Technology, Taipei Medical University, Taipei, Taiwan

[2]Institute of Biotechnology and Pharmaceutical Research, National Health Research Institutes, Miaoli, Taiwan

[3]Institute of Bioinformatics and Systems Biology, National Chiao Tung University, Hsinchu, Taiwan

[4]TIGP-Bioinformatics, Institute of Information Science, Academia Sinica, Taipei, Taiwan

[5]Department of Biological Science and Technology, National Chiao Tung University, Hsinchu, Taiwan

[*]Corresponding authors.

* E-mail: tsuanhsu@nhri.org.tw (JTAH); moon@faculty.nctu.edu.tw (JMY)

[◎]These authors equally contributed to this work.

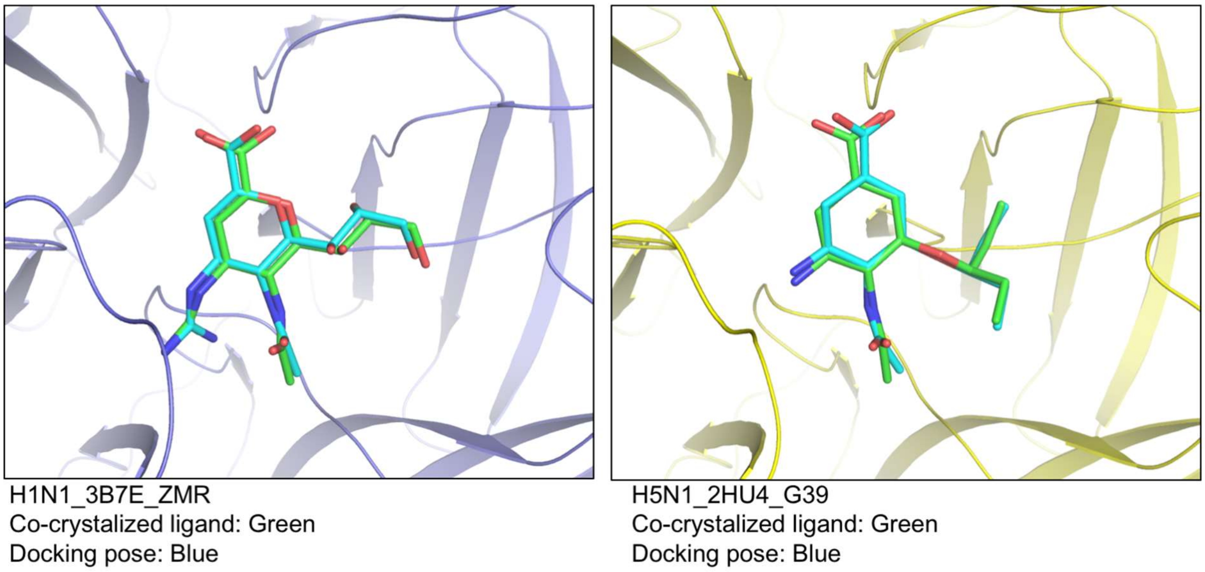

Figure S1. Docking validations using pose selection. The co-crystallized ligand for H1N1 (PDB ID: 3B7E) and H5N1 (PDB ID: 2HU4) was docked (blue) and superimposed with their co-crystallized (green) structure.

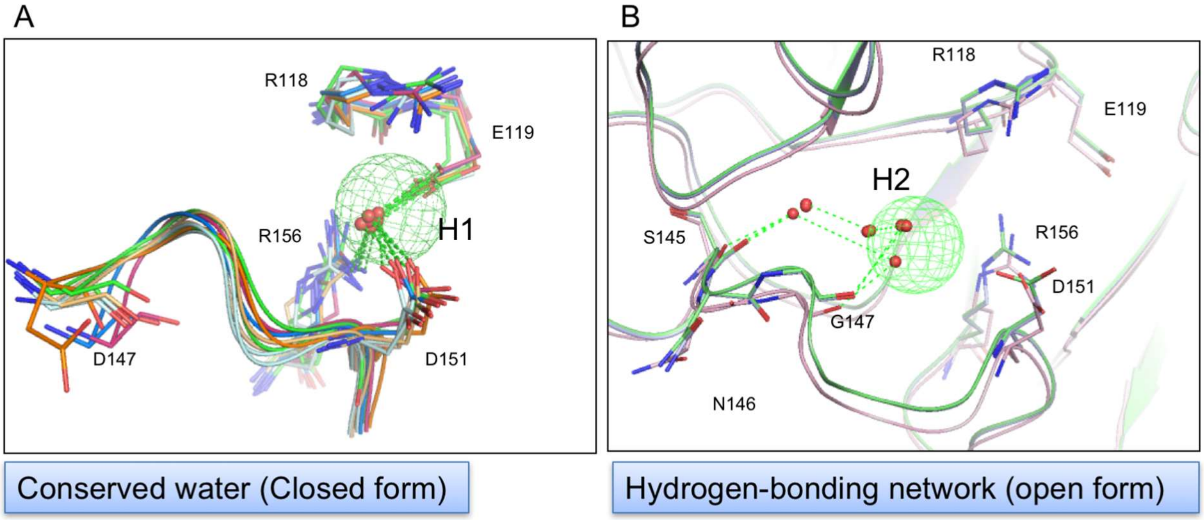

Figure S2. Conserved hydrogen-bonding interactions between water atoms, H2 and H3 anchors in the 150-cavity. (A) Conserved water atoms observed from the 150-closed form structures in the H1 anchor (PDB codes 3B7E, 1ING, 2AEP, 2HU4, 3CKZ, 2HTW, 1V0Z, 1BJI, 1NCD, 1NSB, and 1B9S). (B) Hydrogen-bonding networks observed from the 150-open form structures (PDB codes 3BEQ and 2HTU) in the H2 anchor. The red spheres indicate the water atoms. The hydrogen bonds are presented by green dash.

Compound ID	Subtype	IC50 (nM)	Compound Structure	H3	E1	V2	V3	V4
GS4071	H1N1<br>H5N1<br>N2	1	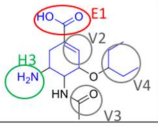	$R{-}NH_2$	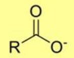	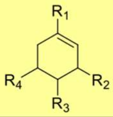		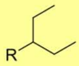
Zanamivir	H1N1<br>H5N1<br>N2		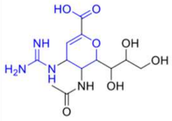	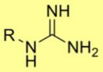	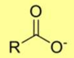	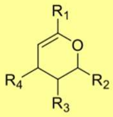		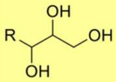
Carbocyclic Analogue 12	H1N1<br>H5N1<br>N2	100	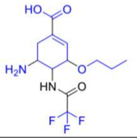	$R{-}NH_2$	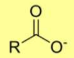	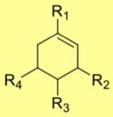	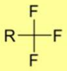	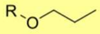
Carbocyclic Analogue 31	H1N1<br>H5N1<br>N2	6300	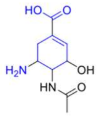	$R{-}NH_2$	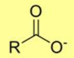	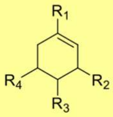		
Benzoic Acid Inhibitor 8	H1N1<br>H5N1<br>N2	15000	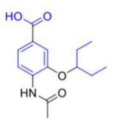		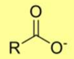	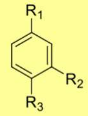		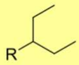

Figure S3. Relationship between anchors and moieties of known inhibitors. The known inhibitors include GS4071, zanamivir, zanamivir analogues, and ATA obtained from BindingDB. The cells are colored yellow and show favorable moiety of inhibitor in the anchors.

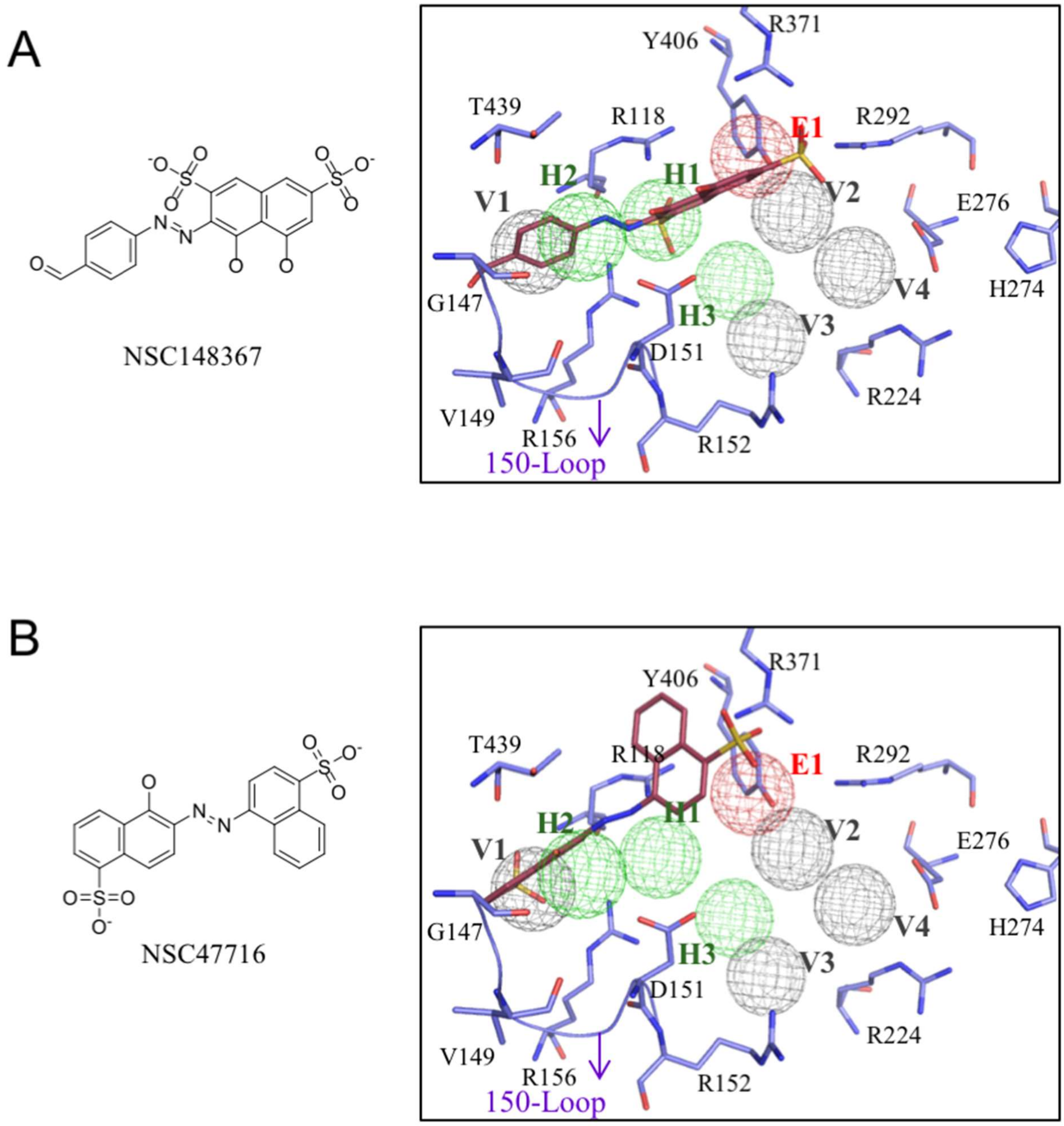

Figure S4. Inhibitor analogues display importance of 150-cavity interaction. 2D structures and 3D docking pose of analogous compounds NSC148367 (A) and NSC47716 (B) listed as shown. The NA active site has a reduced affinity towards the analogous compounds in the 150-cavity site. Furthermore, the compounds did not show sufficient interactions with the sialic site anchors.

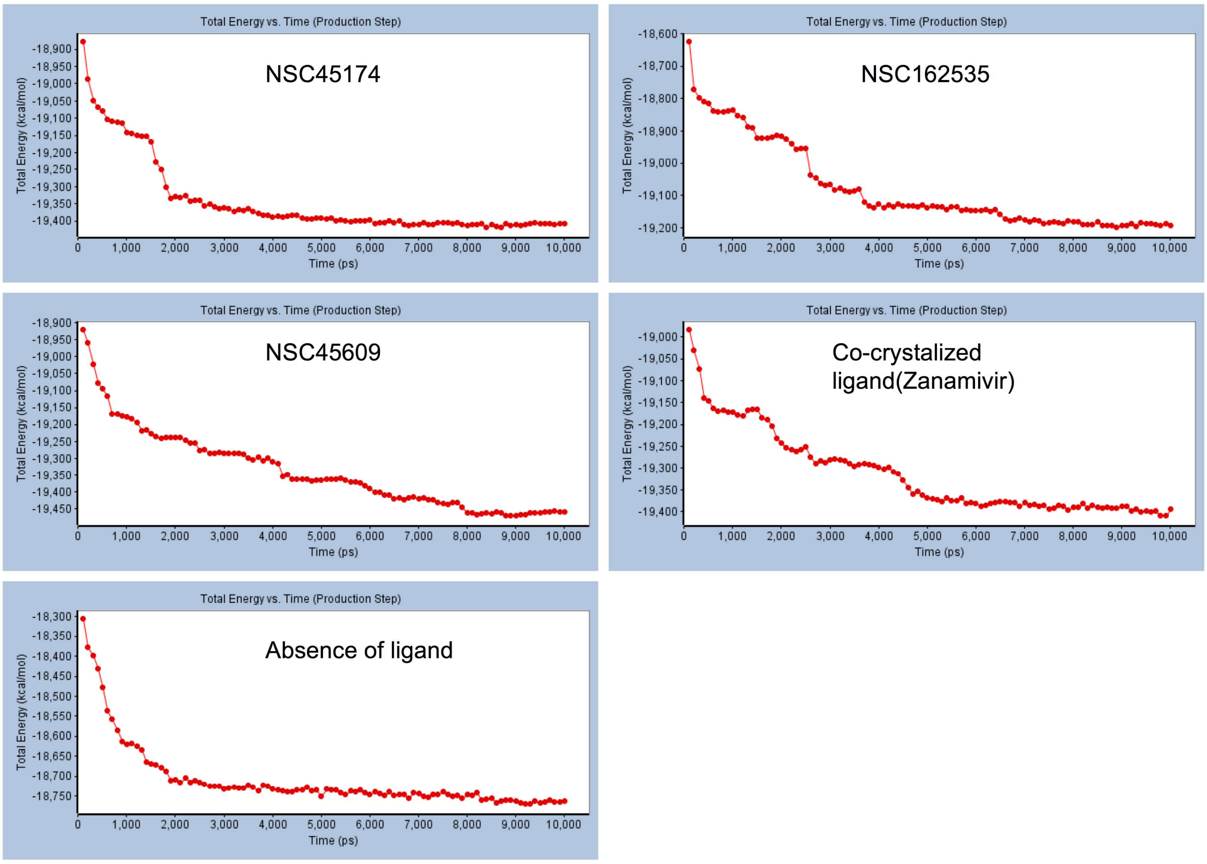

Figure S5**. Total energy from MD simulation.** The total energy from the three identified compounds, co-crystalized ligand (Zanamivir), and absence of ligand was calculated across 10,0000 ps. All structures began to show stable energy at 8,000 ps.

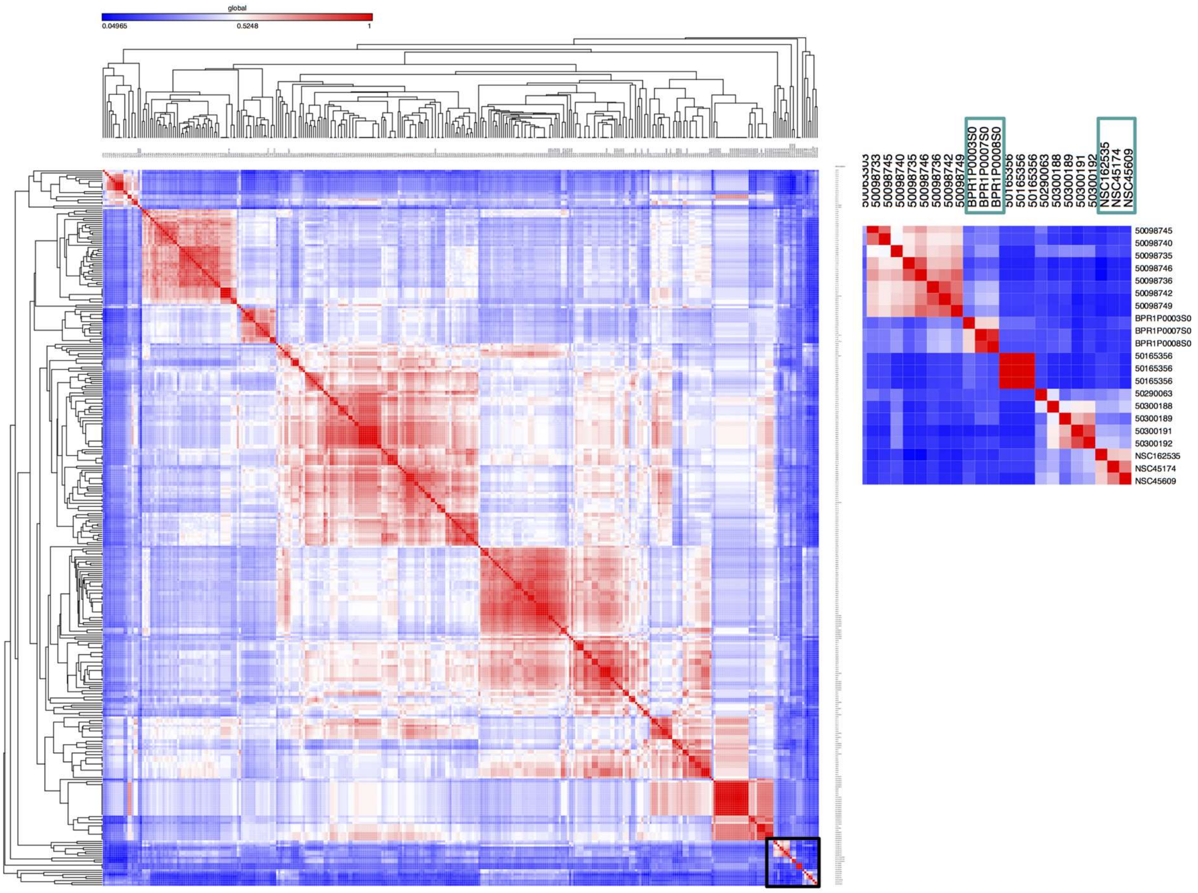

Figure S6. Identified inhibitors have novel structures. A similarity matrix of the three identified inhibitors, the three in-house compounds and compared and clustered to known NA inhibitors from BindingDB. Compounds most similar are colored red, while least similar are colored blue. Black box highlights location of zoomed location. Compound names are listed in zoomed section as shown.

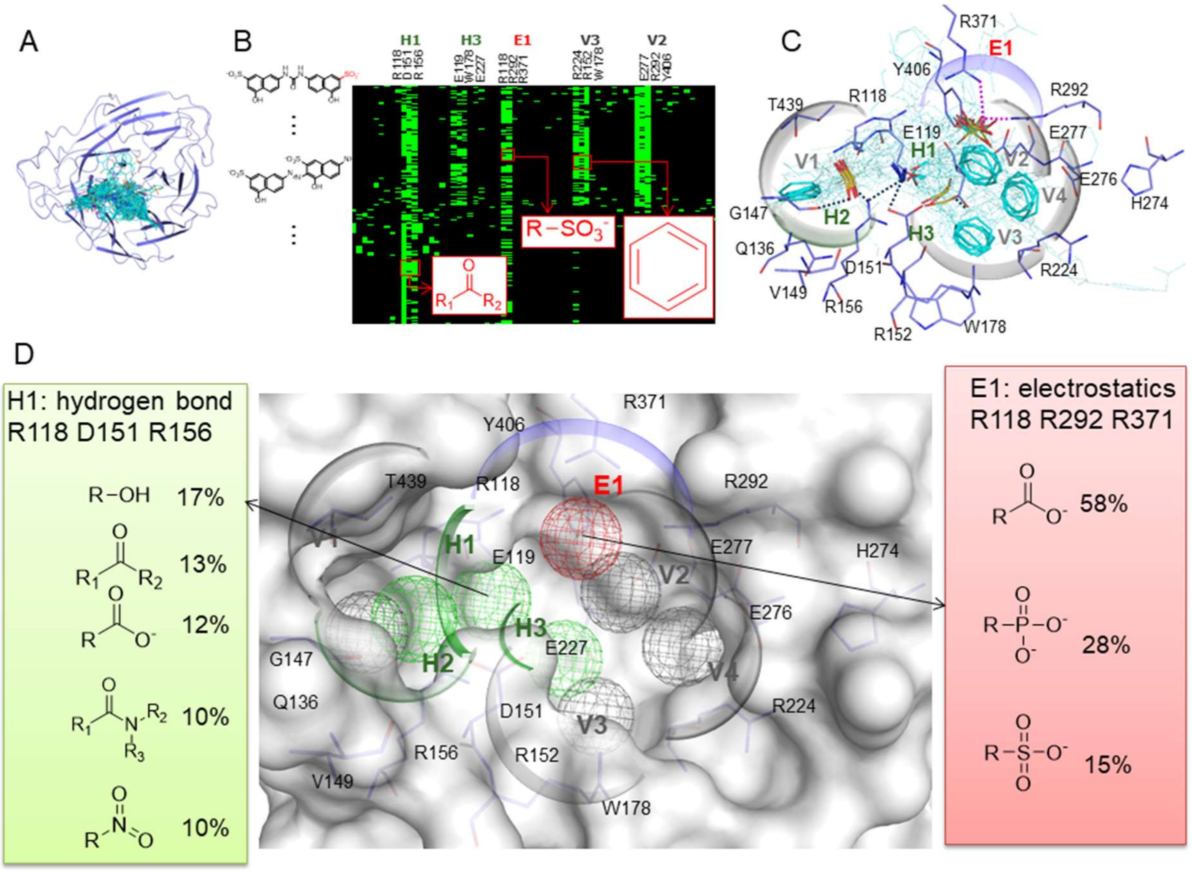

Figure S7. **Main steps for constructing a site-moiety map**. (A) Top-ranked compounds selected by iGEMDOCK. (B) Protein-compound interaction profile for identifying consensus interactions between moieties of screening compounds and residues of pockets. (C) Examples of moieties that consistently interacted with residues of pockets. (D) Site-moiety map of NA using H1N1 NA as the example. The map consisted of eight anchors including conserved interacting residues, moiety preferences, and interaction types. Negatively charged, hydrogen-bonding, and van der Waals anchors are colored red, green, and grey, respectively.
